# Diameter Estimation of Fallopian Tubes Using Visual Sensing

**DOI:** 10.3390/bios11040100

**Published:** 2021-04-01

**Authors:** Amir M. Hajiyavand, Matthew J. Graham, Karl D. Dearn

**Affiliations:** Mechanical Innovation and Tribology Group, Department of Mechanical Engineering, School of Engineering, University of Birmingham, Birmingham B15 2TT, UK; matthewmjggraham@gmail.com (M.J.G.); k.d.dearn@bham.ac.uk (K.D.D.)

**Keywords:** fallopian tube, vision, sensor, diameter estimation, biological tubes

## Abstract

Calculating an accurate diameter of arbitrary vessel-like shapes from 2D images is of great use in various applications within medical and biomedical fields. Understanding the changes in morphological dimensioning of the biological vessels provides a better understanding of their properties and functionality. Estimating the diameter of the tubes is very challenging as the dimensions change continuously along its length. This paper describes a novel algorithm that estimates the diameter of biological tubes with a continuously changing cross-section. The algorithm, evaluated using various controlled images, provides an automated diameter estimation with higher and better accuracy than manual measurements and provides precise information about the diametrical changes along the tube. It is demonstrated that the automated algorithm provides more accurate results in a much shorter time. This methodology has the potential to speed up diagnostic procedures in a wide range of medical fields.

## 1. Introduction

Analysis of tubular structures for biological applications plays crucial roles in diagnosing several pathologies such as cardiovascular disorders, diabetes, spinal stenosis, central retinal vein analysis, and hypertension [1,2,3,4,5,6,7]. All of these applications would benefit from accurate and fast dimensioning for quicker diagnosis [8]. Automatic blood vessel visual extraction tools have higher accuracy and are preferred by practitioners over manual operations. However, due to imperfections, achieving high accuracy for automatic operations can be challenging [9].

Furthermore, they are all difficult to measure using intrusive measuring techniques due to these being biological vessels. It is already standard practice for determining the severity of coronary disease using computer-aided analysis of the vessel profile. Coronary arteriography estimates the dimensions, hemodynamic resistance, and atheroma mass of coronary artery lesions [6,10,11,12,13].

A wide range of methods is used to extract and enhance dimensional and topological data, measured from biological vessels in medical images [14,15,16]. They all compete in particular aspects such as computational costs, robustness, and imaging modalities [3,4,17,18]. Although these methods are beneficial for vessels and manual measurement [19], they pose several problems for general use and accurate diameter processing due to human inconsistency and inefficiency.

There are specially developed algorithms designed for specific vessel types, usually comprising Gaussian elongated linear filter kernels that create a response to specific vessel types in certain orientations. The specific application determines the kernel filter. There is, however, a higher probability of receiving false responses if kernels are employed for a more general purpose.

These filters have difficulty fitting non-standard vessels that are highly twisted or irregularities in diameter that could be vital to detect for diagnosis [17,18]. Furthermore, they also are unable to factor in the wide variety of noise features and the intensity spectrum found in the real-life images. Manniesing et al. describe high-level segmentation types that could affect the vessel measurement’s final diameter [20].

Various studies have focused on analysing specific regular shapes’ dimensions using different conventional techniques, such as using perspective projections, to determine boxes’ geometry. However, these methods do not detect arbitrarily shaped objects [21]. Machine learning techniques produce similar outputs focusing on feature recognition and point mapping. While these techniques produce accurate measurements between predetermined points, they are inherently inflexible for use with unfamiliar vassal structures due to the non-adaptability of the recognition tools and the need for the vast amount of training data [22].

Other feature recognition algorithms are equally inflexible due to their need to map the structure mathematically. Although these methods can produce accurate dimensions of particular objects, a curve’s parameterisation can over-look tiny details and simplify the actual shapes. Furthermore, mathematically modelling the object limits the algorithms’ complexity and diversity attempt to dimension [23,24].

There are various techniques to extract the centrelines of the objects and hence the biological tube diameter. Using voxel images to create centrelines in three dimensions directly provides higher accuracy in results. However, these 3D images are usually generated using costly techniques and are highly challenging in medical applications due to the operational procedures. Therefore, any voxel-based method is not appropriate for medical applications, as most of the images are only captured in two dimensions [25]. A distance transform can be used to produce accurate centrelines of complex shapes, even faster than our proposed thinning technique. However, this method cannot guarantee a continuous non-branching centreline path. The distance transform’s nature produces small branches and even makes ‘hole’ artefacts in line with a secondary thinness check. This paper’s centrelines need to be connected by two neighbouring pixels to produce a reasonable length index in our application. Furthermore, through parallelisation, our proposed thinning method’s speed can be similar to the distance transform [26].

More detailed segmentation to recreate a 3-dimensional centreline extraction has been attempted with relative success, with up to 93% accuracy compared to the original 3d vessels [19,27]. Neglecting, however, potential 3-dimensional elements of vessels has been shown to affect the accuracy of the diameter measurement by less than 1% [28].

There have been advances in this problem’s edge detection aspects [29], including the creation of high-resolution masks for width checking. However, the specific nature of these techniques means they cannot be used for a general-purpose algorithm. Furthermore, these masks can be deployed at angles to the centreline to determine a tube’s diameter. This method creates an approximation for the diameter and potentially adds to the processing time required to fit the mask. Segmentation has been possible with neural networks with excellent results [30]; however, due to the nature of training, it can only be used on data sets that are very familiar to the trained sets, such as fundus images. Any application that requires more varied or general data sets would struggle to get any results or any scaled results.

Many studies model the vessels’ tortuosity by polynomial fitting techniques [1,31,32]. These algorithms increase the error in centreline estimation depending on the vessel’s complexity, reducing accuracy if used for diameter measurement. Furthermore, the polynomial order is greatly affected by the type of vessel measured, making this method unsuitable for general purposes. The tramline effect caused by the lighting on the veins can enhance other algorithms [33]. This effect varies, however, with different ambient lighting and the structure of the vessel itself. Similar routines have been successful, but the centre and normal lines’ extraction is not optimised, enabling only a vein-type classification. The algorithm proposed in this paper is inspired by this method to create an accurate diameter detector [34].

Estimating blood vessels’ diameter or other biological tubes play crucial roles in various diagnostic and therapeutic medical applications. However, accurate measurements are not feasible using manual measurements as the diameter changes along the vessels’ length. This paper describes the development and success of a novel real-time method for width extraction of any arbitrary biological tube by employing an automated estimation solution. The algorithm extracts a diameter along the entire length of any tube using a single image, where the camera’s visual field maintains the object’s aspect. Each frame is optimised for maximum performance through a pre-processing stage; the method then extracts the edges and creates a binary shadow of the image.

This paper contains three main sections; the first provides the details of the pre-possessing, edge detection and binarisation of the algorithm. Then the width finding process from a binary image is discussed. This paper concludes by demonstrating and validating the algorithm.

## 2. Materials and Methods

**Figure 1:**
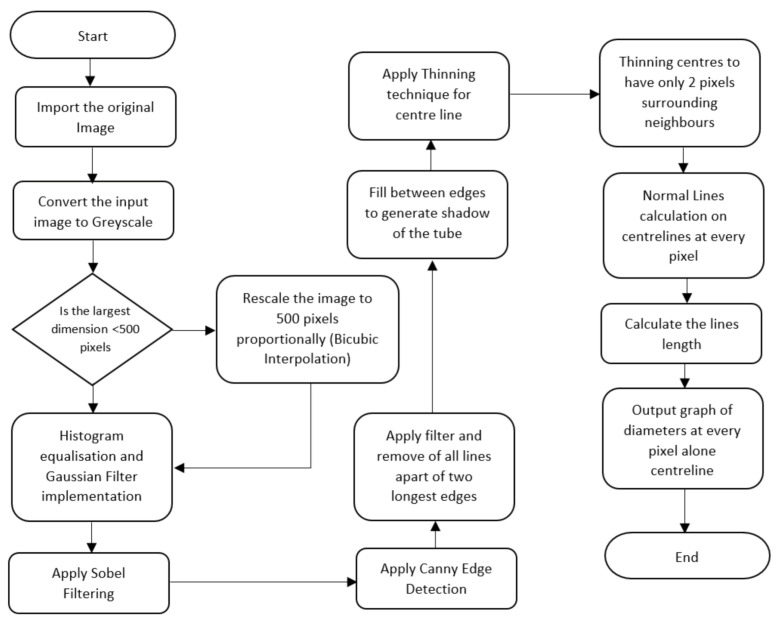
Operational flowchart of the algorithm.

The developed algorithm detects and then measures the dimensions of biological tubes from diagnostic images. The detection technique is divided into three stages: pre-processing, feature extraction and diameter measurement, shown in the flowchart in Figure 1. Before analysis, however, the raw images are optimised for extraction to increase the detection’s effectiveness.

### 2.1. Pre-Processing

The initial operations are performed on the raw input image defined as *Iin*. This pre-processing stage converts the Iin into a greyscale image, convolves it with a Gaussian kernel, and then performs a histogram equalisation to increase the contrast definition. This stage aims to convert generic raw input images into high contrast, low noise format with minimal information loss, thus maximising the data’s quality to be analysed by the width processing algorithm.

The standard colour model for digital coloured images is a 3-dimensional matrix and a 3-channel dimension RGB, representing red, green and blue, with the value of channels representing the colour for each image. A greyscale image is 2-dimensional and has an intensity range between black and white. Each component’s level is equalled in a grayscale image by averaging the RGB within each pixel, defined in (1). This equation can also be manipulated to give more substantial weights to colour channels over-represented in the vessel that is being analysed.

(1) $$GrayScale_{intensity}=\frac{R\left(x,\;y\right)+G\left(x,\;y\right)+B\left(x,\;y\right)}{3}$$

The image is a 2-dimensional matrix, allowing the dynamic range to be analysed and increased. The input image, *I*(*x*,*y*), will rarely be noiseless, resulting in many more edges being falsely detected and non-continuous gradients at the edge pixels resulting in gaps on the edge lines. A 2-dimensional scaled Gaussian smoothing kernel eliminates false detection and is convolved across *I*(*x*,*y*) as demonstrated in (2).

(2) $$G_{c}\left(x,y\right)=\;\frac{1}{2\pi \sigma^{2}}e^{-\frac{x^{2}+y^{2}}{2\sigma^{2}}}\;$$

where *x* and *y* are the coordinates in 2-dimensions along the Gaussian distribution curve, this kernel performs a weighted dot product, averaging the convolved pixel with its neighbour. The x-y distance of a single-pixel from the central pixel determines the contribution to the final pixel value, then following a dot product with the corresponding value on a 2-dimensional Gaussian distribution. This distribution can be used for any size of an image by increasing its *σ*, which increases the weight neighbouring pixels have on the average.

The luminosity distribution must be manipulated in order to maximise contrast at the edges of the tubes. No assumptions can be made about the distribution of luminosities or the local contrast in interesting areas, such as tube edges. Therefore, the ideal histogram of luminosities for an image *I*(*x*,*y*) is an entirely equalised distribution. This histogram gives the maximum average dynamic range of the image. Equation (3) defines *cdf*(*j*), the ideal situation based on a cumulative distribution function (i.e., the cumulative occurrences of the luminosities at each value, *j*, where *n* is the number of occurrences). Equation (3) defines the cumulative distribution function, *cdf*. Calculating the cumulative occurrences of each intensity *n*, between 0 and 255 (for an 8-bit greyscale image) where *c_i_* is the number pixels at that intensity level and c is total pixel count.

(3) $$cdf\left(n\right)=\sum_{i=0}^{n}\frac{C_{i}}{c}$$

The intensity range of an 8-bit greyscale image is within 0 to 255. In (4), *E_f_* is a multiplication factor that optimises the dynamic range, where *n* represents the number of bits for the greyscale image, and the denominator is the dynamic range.

(4) $$E_{f}=\frac{255}{Grey_{Max}-Grey_{min}}\;\;\;\;\;,\;\;\;h\left(f\right)=\left(g\left(f\right)-grey_{min}\right)\left(E_{f}\right)$$

Equation (4) increases the dynamic range of the intensities to encompass the 8-bit structure fully but does not affect the intensities’ distribution. The standard deviation and variance, hence, remain identical to the original image. A limitation of this method is that it does not increase contrast in critical edge areas. In images with a high dynamic range but a low standard deviation, edge detection can be difficult. Consequently, the histogram equalisation method enhances the image and eliminates the edge detection problem.

The new histogram of intensities, *Bhist*, is calculated in (5) using each intensity value distribution and mapped to new channels of equal size over the entire dynamic range, with a maximum value, *L*.

(5) $$B_{hist}\left(n\right)=round\left[\left(L-1\right)cdf\left(n\right)\right]$$

This new matrix can now be compared, index by index, with the original intensity array, *n*, creating a conversion table and critically replacing the image *I*(*x*,*y*) luminosities, from *n*(*n*) to *B_hist_*(*n*). Edge detection is, therefore, dramatically increased in poorly defined images as a result of the nonlinear increase, in contrast, focusing on the intensities with the lowest contrast.

### 2.2. Width Finding

After pre-processing the images, the algorithm detects the diameter of the arbitrary vessel. To find the vessel’s width along the tube, the processed image, I, is passed through a Canny edge detector and sorted for noise outputting only the vessel’s edges. The image is then binarised and filled with a bounded region to form the vessel shape’s solid binary silhouette. A centreline of the vessel will then be extracted between the extracted edges. It is then binaries, fills to form a solid binary silhouette and a centreline of the vessel. Lines are calculated normal to the centreline at every pixel distance until it encounters the vessel’s edge. Consequently, the length of these lines is determined to calculate the diameter at every single point along the tube.

#### 2.2.1. Edge Detection

An edge pixel is the maximal magnitude of a grayscale image’s intensity matrix (Known as Matrix I). To deduce the rate of change of the image’s intensity, two gradient matrices of the image, *Ix* and *Iy*, are calculated through a convolution of the 3 × 3 Sobel operator’s in (6) with the greyscale pre-processed image, I. This operation produces two matrices highlighting the high spatial frequency regions corresponding to the orthogonal edges.

(6) $$Sobel\;X=\left[\begin{array}{ccc} -2 & 0 & +2\\ -3 & 0 & +3\\ -2 & 0 & +2 \end{array}\right],\;Sobel\;Y=\left[\begin{array}{ccc} -2 & -3 & -2\\ 0 & 0 & 0\\ 2 & 3 & 2 \end{array}\right]$$

The Sobel operators estimate the 2-dimensional gradient from the magnitude of the two orthogonal vectors. The magnitude of the gradient is the product of the two matrices through (7), similarly the orientation of the gradient, θ, is determined at every pixel using (8) and is a bearing angle from the positive *y*-axis travelling clockwise [35]:

(7) $$\left|I\right|=\;\sqrt{I_{x}{}^{2}+I_{y}{}^{2}}$$

(8) $$\theta =\arctan\left(\frac{I_{y}}{I_{x}}\right)$$

**Figure 2:**
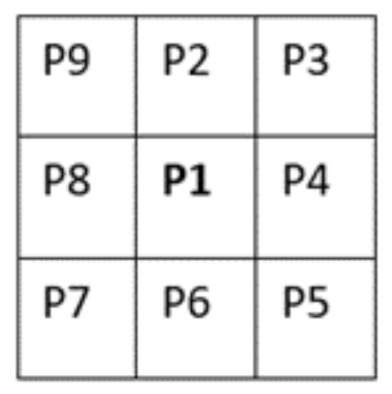
Schematic view of the edge detection operation.

Orientation angles simplified with intercardinal precision are the preferred format when finding pixels that have parallel gradients with each other and calculate eliminating non-maxima gradients (i.e., those that are not the peak of the edge). Table 1 shows the rounding logic and Figure 2 is the illustration of the location key of each pixel considered.

In each examination, only the detected edges’ maxima remain within the matrix denoted as I, leaving a matrix of maximum magnitude gradients of all detected regions with increasing intensity.

Unnecessary noise is removed from the matrix using the double thresholding Canny edge detection method [36], enhancing the algorithm during object segmentation [37]. The examination checks every pixel’s intensity level against two defined threshold values, given as z1 and z2, representing the image in binary, while prioritising the most robust detected edges while not ignoring weak signals of potential edges. For example, if a pixel in Matrix I is more significant than z2, then the pixel in the new binary image, BI, will be set to 1. If the pixel is smaller than the value of z1, then the binary image pixel will be set to 0. However, if the pixel’s value is between z1 and z2, this can be either 1 or 0 based on a branching method.

Following the branching method, non-zero pixels detected from the eight neighbouring pixels are stored and flagged in a new array, denoted as A(pixel). A check on random neighbouring pixels in the new array looks for non-zero value pixels which are not already in the array are stored in new positions in the array. This process repeats at every index of the array. The branching scan stops if the intensity of one of the pixels on the branch is higher than z2, setting the original pixel to 1 in the binary image. The branching can also stop if no other non-zero pixels are detected, setting the original pixel to 0. This strategy ensures that every non-zero pixel linked to the initial pixel is considered in calculating an edge’s intensity, identifying minor gradient change that is part of a significant edge in full.

The output from this method is a binary image of detected edges. The algorithm assumes that the most extended two edges detected are the edges for the tube used for width finding and relevant intended body. It converts edges into lines, then into a data structure that stores each line as an individual variable, sorting the structure for the most extended entries and deleting all but the two longest lines. The final image will only display the information of the desired edge. There is a manual override feature to segment the vessel’s required edges if this should be required.

#### 2.2.2. Filling

A thinning algorithm creates a silhouette of the original object to extract the centre line between the edges and define the object’s entirety. This algorithm during filling of an image assumes:

1. Extremities of the vessel are outside the image scope BI, or the entire vessel is enclosed in the image;
2. Only two continuous edges are detected from the tube left in the image.

The Binary Image matrix BI (X,: ) considers each column individually. y1 and y2 denote non-zero values of the initial and final pixels, they-coordinate in each column. If pixels satisfy y1 ≤ y ≤ y2 within the matrix, the algorithm assigns a value of 1 to fill the intermittent pixels. This process repeats for each row BI (: Y), filling any object defined in this orientation. The limitations of this approach are:

1. There are partial areas of the vessel detected, resulting in single line detection;
2. Regions of a vessel where neither top nor bottom of a line is present;
3. There are vessel areas where one of the edges has moved out of the scope of the image.

The communitive nature of the binary line mitigates these errors. For point 1, the edge-pixel(s) in the adjacent column to the previous edge-pixel will always have an equal or consecutive index to the previous column, interpolating missing lines. In the case of a lack of line detections, the initial and final y is equal.

Consequently, to compensate for this, an edge with no data can be estimated by testing and comparing the new y value with the previous columns y1 and y2 using (9). The undetected edge acts as the second index for the current column of filling.

(9) $$y_{new}\equiv \;y_{i}\;\cup \;y_{new}\equiv y_{i}+1\;\;\cup \;y_{new}\equiv \;y_{i}-1$$

For point 2, where both the top and bottom pixels in the column are absent, the programme implements a similar routine. In this case, there is no data point detected in the column, and (9) yields y1 and y2. Finally, for point 3, which is an exceptional condition of case 1, the same process is applied except y1 and y2, the maximum or minimum index value.

However, they could be interpolated further with columns that occur later in the matrix, still to be analysed. A linear interpolation filling for the missing data is conducted by creating a Bresenham line between the missing data points and then filled. The severity of the missing edges and inaccuracy was ±1.5 pixels during the algorithm’s testing, considering the missing data’s median length was 1 pixel long. Increasing the accuracy of the interpolation model is limited by the restricted propagation distance.

#### 2.2.3. Centreline Extraction

Surface imperfections on lines normal to edges prevent the discovery of the actual diameter. To locate two complementary sides requires further mathematical analysis from the centroid axis. Consequently, the line which is normal to the centroid axis in the object is defined to find the width.

This operation requires a binary morphological method of thinning that has no bias to orientation, produces no branches and has a relatively fast processing time. The Zhang Suen algorithm [38] provides a fast and unbiased centreline extraction, revealing the binary ‘shadow’ image’s exact centre. This image skeleton extraction method consists of removing all contour points in the image except those skeleton points. This method creates a centreline too ‘thick’ for simple applications as some pixels have three direct neighbours but adds ambiguity to create a 1-dimensional array of coordinates along the line.

**Figure 3:**
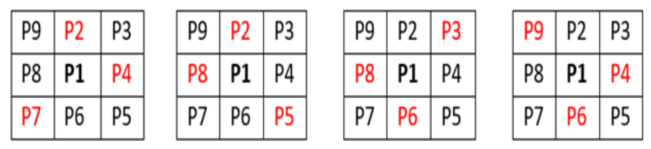
The second thinning operation and operational logic.

A second thinning operation is required, at every point along the centreline, checking pixel P1 for more than two neighbouring pixels. If this was true, then the logic of the image was calculated. This operation makes BI (P1) = 0 if the corner pixel and the pixels opposite to it in the two complimentary cardinal directions are one and create an exhaustive list of situations where P1 are 0, this logic is visually shown in Figure 3.

Under these conditions, the centreline BI thins to become a line of a consistent pixel neighbour count of 2. This thinning is a crucial step, representing the line as a one-dimensional list of coordinates with each point on the line assigned a specific index.

Concentricity, defined as the absolute midpoint between the two edges, can be tested by comparing the difference between the radii of circles subtending centrally from each edge on the line. This definition is non-absolute as surface irregularities under this definition would shift the centreline from the centroid axis. However, within tolerance, this definition holds some truth.

#### 2.2.4. Normal Line Extraction

Finite difference approximations estimate the gradient at every point along the arbitrary centreline rather than a polynomial line to reduce fitting line errors. These occur due to the variety of forms the centreline can take to suit any single line fitting algorithm. Additionally, these errors compound when prevented from replacing existing edges of the approximated line.

An accurate estimation of the curve’s gradient derived from the finite difference approximation applied over a greater distance and accounting for local gradient variations from pixel to pixel is illustrated in (10). The distance used was five pixels along each side of the target pixel, mitigating errors induced from the image’s pixilation.

(10) $$grad\left(i\right)≅\;\frac{\left(y_{i-4}-y_{i+4}\right)}{\left(x_{i-4}-x_{i+4}\right)}$$

The gradient and origin of a new straight line, normal to every pixel along the centre-line, derives from the gradient’s negative reciprocal at every line index. This straight line is then converted into a Bresenham line [39] to map the theoretical line into a raster form drawn on the image, with a secondary function of finding collisions with the filled image’s edge. The line was drawn onto the ‘shadow’ binary image, BI until the next pixel on the path of the rasterised Bresenham line is on a position with the value of 0. Thus, it terminates at the contour of the object’s shadow. This calculation transpires in both directions perpendicular to the centreline.

The output from these calculations is a one-dimensional array with an index containing pixel coordinates of the terminating points of two Bresenham lines along the vessel’s centreline. Equation (11) calculates the Euclidean distance, *d*, from each other. These diameters can be plotted against the pixel index on the centreline, showing the diameter of an arbitrary vessel at every pixel along its centroid axis [40].

(11) $$d_{i}=\;\sqrt{{\left(x_{\left(i,1\right)}+x_{\left(i,2\right)}\right)}^{2}+{\left(y_{\left(i,1\right)}+y_{\left(i,2\right)}\right)}^{2}}$$

## 3. Results

### 3.1. Operational Results

In this paper, a conventional image processing approach extracts contour information of the fallopian tubes for diagnostic purposes. As the tubes are of an irregular shape, the algorithm detects straight, inclined and curved geometry. This combination confirms the algorithm’s ability to detect with high accuracy the geometry of the fallopian tube.

**Figure 4:**
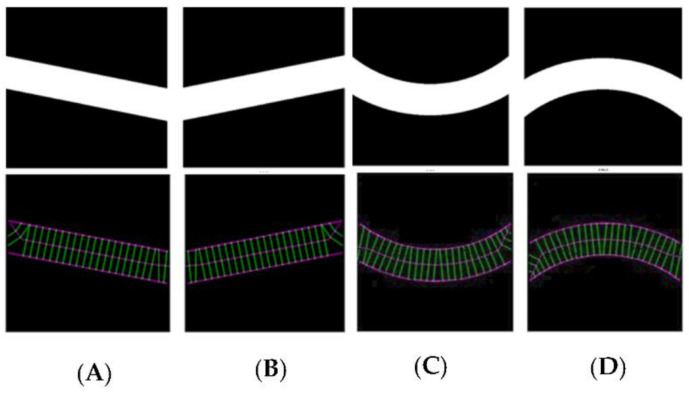
Test and processed images for operational validation of the algorithm. (**A**,**B**) are positive and negative 100-pixel lines at a 10 degree inclined, (**C**,**D**) are positive and negative 500-pixel segmented wide arc of 1000 pixel outer curvature diameter annulus.

Control images were introduced to the algorithm to generate evaluation data for straight tubes with a 10° inclination angle and curved tubes on a 500-pixel radius arc. Generated data illustrate the algorithm’s performance capability for different image geometries and sizes, demonstrating algorithm repeatability and orientation independence. Figure 4 shows all the images (500 × 500 pixels).

**Figure 5:**
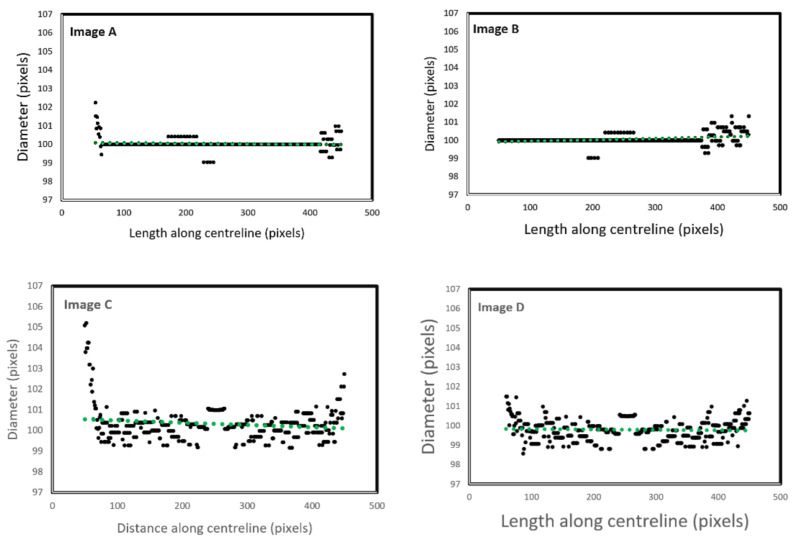
Diameter estimation of the test images. The green dots represent the overall directions of the data and the distributing pattern in each image, (**A**,**B**) are positive and negative at a 10 degree inclined, (**C**,**D**) are positive and negative.

Figure 5 illustrates the automatic visual measurements of the tubal diameters among the controlled images. Small noises oscillate at the images’ extremes due to the thinning algorithm’s initiation, at points where the object edges are undefined. The algorithm thins the image wall and the edges causing initial centreline extraction errors, then rectifies after the entire vessel definition is within the image. Interpolation could partially rectify these faults. This approach would be unsuitable for a large variety of vessels as any possibility of edge positions could be outside the image, adding uncertainty. It is more accurate to leave the algorithm in its current state, analysing only the provided data, giving high accuracy of 1-pixel error in the interest areas within the domain of (75, 500) pixels.

**Figure 6:**
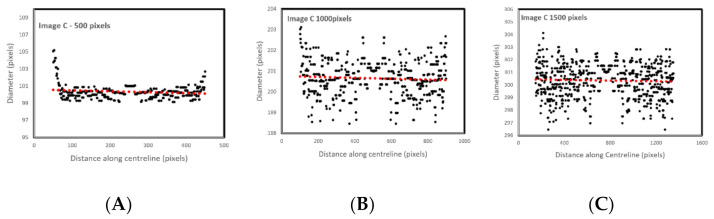
The effect of resolution on diameter estimation accuracy for the straight tubes with a 10° inclination angle. (**A**) 500 pixels wide, (**B**) 1000 pixels wide and (**C**) 1500 pixels wide.

The results given in image A and B are for the straight tubes with a 10° inclination angle. However, for the 500-pixel radii tubes, the data are noisy with higher fluctuations. The accuracy, however, remains the same (less than 1 pixel). This increase in fluctuation could be due to the image’s pixilation due to estimated edges and centreline to the nearest pixel. The placement of these pixels is not perfect in representing the continuous form of the circle. The over and underestimation of these values further supports this conclusion—the diameter averages 100.1 and 99.9 pixels in image C and D, respectively. Errors would reduce with increasing image resolution. Figure 6 shows the effect of image resolution on diameter estimation accuracy (based on the noisy signals shown in Figure 5C). Accuracy reduces with image resolution. In the images, the red line trends are aligned to the actual diameters. However, the total report error for the 1500 pixels is 3%, which makes the algorithm reliable even for bigger size images.

**Figure 7:**
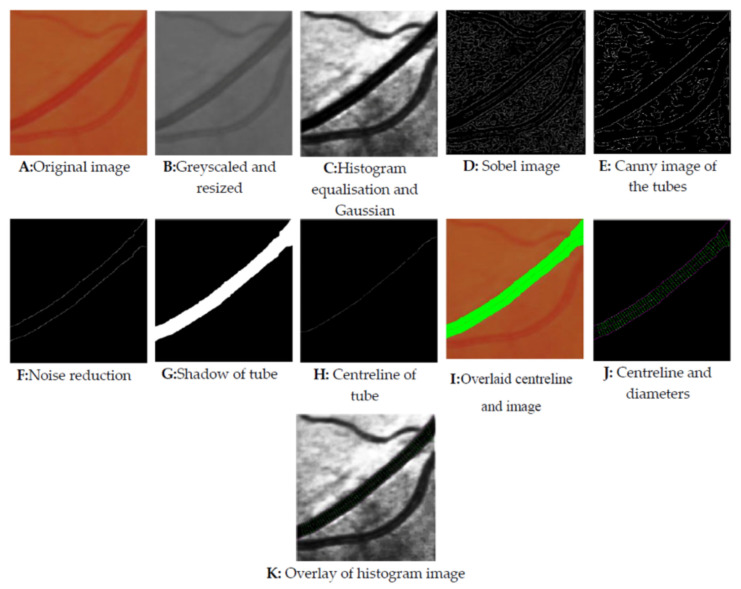
Extracted vein diameter using the developed algorithm.

Images obtained from the literature were employed to demonstrate the image-processing steps and evaluate the algorithm efficacy regarding real (rather than simulated) biological tube tracking, including diameter estimation for real biological tubes. Figure 7 illustrates a step-by-step procedure of extracting the biological tube’s diameter using vessel images harvested from Reference [1]. In essence, the algorithm converts the RGB image [A] to greyscale [B] immediately on receipt. Then the histogram equalisation [C] is applied to improve the dynamic range of the image. The Sobel [D] and Canny [E] images follow finding all potential edges on the image, further processed using a noise reduction [F] technique to eliminate lower quality edges in the image (such as those too short or ill-defined). Sobel Operator is employed as a filter to extract the edges of the vessels, and a Canny edge detector is used to minimise the noises of the processed images further. Then a centreline is thinned [H] from the shadow of the vessel [G]. Overlaying the centreline and image shadow [I] on the original image demonstrates the algorithm’s accuracy. Applying the histogram image overlay [K] improves the edges’ visibility, including the centre line and diameter [J] evaluation lines. The algorithm finally outputs a diameter estimation (As the tube’s diameter is non-uniform along the tube).

**Figure 8:**
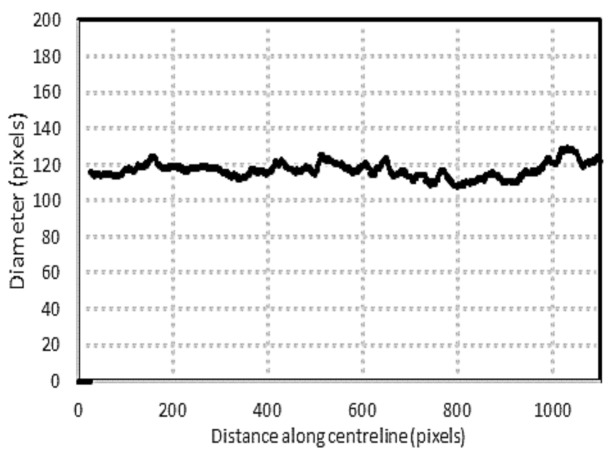
Extracted vein diameter using the developed algorithm.

Figure 8 shows the measurement of the extracted vein diameter using the algorithm. The average measured diameter is 115.91 ± 7.15 pixels (mean ± SD). The variation from the average is approximately 6%. Visual confirmation of the images illustrates the accuracy of the algorithm in detecting the diameter of the tube.

### 3.2. Application Results

Following confirmation of accurate performance using biological images, the algorithm examined a set of in vitro fallopian tube images. The fallopian tubes are represented as biological tubes with arbitrary contours along their length, making the accurate measurement of the diameter very challenging. Nevertheless, a deep understanding of fallopian tubes’ real diameter will enable practitioners to understand these tubes’ fundamental geometry better. For this test, sheep fallopian tubes represent a reliable representative of human models. The fallopian tubes were stored in phosphate buffer solution at 37 °C and transferred to laboratories within an hour after collection. Samples were trimmed with excess ligaments separated where feasible and photographed using a 12 MP camera with background lighting. The images show the tube’s random positioning over a glass slide, lighting from behind, covering the distal to the tube’s proximal sections. The algorithm detects the fallopian tube accurately, despite signal noise within the images caused by excess unremoved ligaments. Diameter estimation and contours from the algorithm indicate good accuracy when overlaid on the original images.

**Figure 9:**
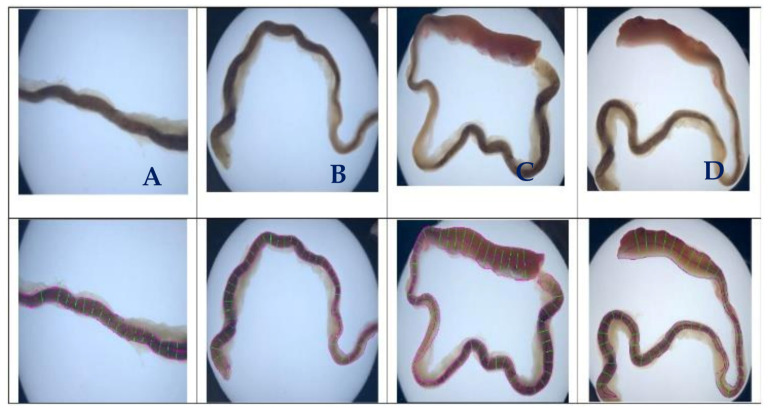
Sheep fallopian tube (original images and processed images—for visual confirmation of the accurate estimations).

Images were imported to the developed algorithm and used for manual measurements using ImageJ software (Version 1.52). Figure 9 shows the results of the diameter estimation of the fallopian tubes. These over layered images confirm accurate estimations of the diameter, and the random positioning of the tubes illustrates the ability of the developed algorithm to detect arbitrary shapes of the biological tubes.

**Figure 10:**
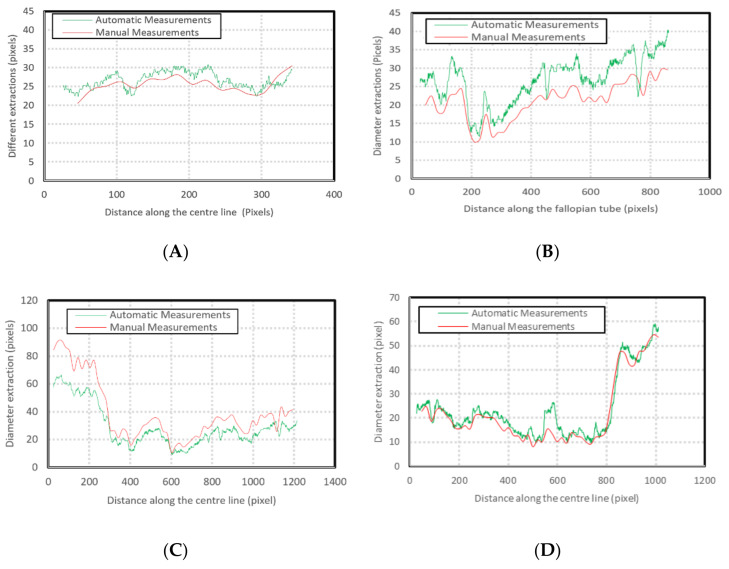
Extracted vein diameter using the developed algorithm.

Additionally, some other ligaments were attached to the primary fallopian tubes within the images, impacting detection accuracy. However, the novel developed algorithm minimised these effects. The algorithm demonstrates a robust estimation considering existing systemic noise, such as other attached ligaments. Figure 10 compares the manual and automatic measurements. The variations are due to human error in detecting the appropriate pixel during manual measurements.

The existence of non-related structures is unavoidable when evaluating biological tubes visually. These can be in the form of soft tissues and other types of ligaments attached, in the case of this paper, to the periphery of the fallopian tube. In addition to these, and unlike other biological vessels, the diameter and wall thickness vary within the tube. All of these sources of noise have been eliminated during visual processing. This paper’s images have been obtained under laboratory conditions, reducing noise present in clinical imaging procedures.

This study focuses on extracting the external contour of the fallopian tube. Existing noise within the image is eliminated during the processing stage. However, the current algorithm would require enhancement to be utilised in other clinical imaging procedures (i.e., ultrasound or X-ray) and capture internal diameters from X-ray images. The approach described in this paper has some restrictions if used in ultrasound as various layers are required, and the size of fallopian tubes and the existence of other ligaments increase challenges to the clarity of generated images.

The comparison between the manual and automated measurements was presented in both millimetres and pixels as the pixel size varies between images. There is a considerable amount of human error in manual estimation affecting the result accuracy. This error may manifest as the differences observed between manual and automated measurements—the result of automated detection for the test images are given in pixels.

The fallopian tubes’ diameter translates to the metric data by calibrating the pixels, including the pixel size using a reference in the images, all validated using ImageJ. The manual measurements were conducted on ten different points within three main sections of the tube in each image. Table 2 shows the total number of readings within each image. The results are illustrated in pixels to demonstrate the accuracy of the measurements. As the results indicate, the average of the differences is less than 1 mm.

**Figure 11:**
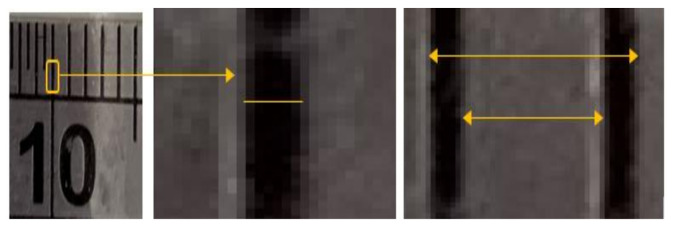
Extracted vein diameter using the developed algorithm.

The variations mentioned above indicate the differences between manual and automatic measurements. During calibration, the manual reading accuracy varies between the users adding inaccuracy and inconsistency to the measurements. Even the measuring of a single division of the reference indicates more than 17 pixels of variation. Each division contains 26 to 43 pixels, increasing manual measurements inaccuracy as each pixel can be manually selected. A small number of readings also decrease the accuracy of the correct diameter extractions for arbitrary biological tubes. As shown in Figure 11, each line of the division contains almost 8 pixels. On the other hand, each division which is 1 mm, is between 27 and 43 pixels. In this particular measurement, the algorithm automatically selects a pixel size of 35 pixels/mm.

The tubal diameter continuously varies along the length of the tube. This variation makes manual diameter estimation very challenging, suggesting inaccuracy and inconsistency in measurements. Increasing the number of measurements increases accuracy; however, increasing the number of manual readings is very time-consuming. A further image of the fallopian tube was considered to find the estimated diameter based on a reference in the image. In this measurement, the data were automatically extracted from the algorithm using the reference existing in the image.

**Figure 12:**
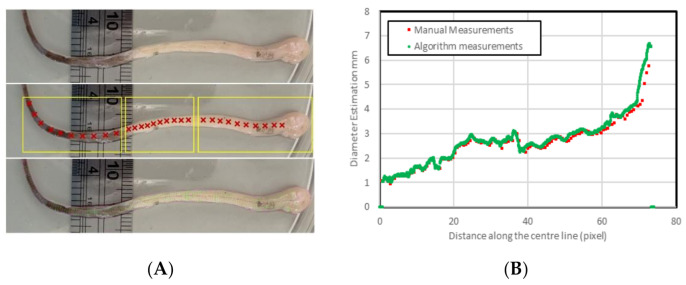
Comparison of the manual measurements versus automated measurements using a higher number of reads, (**A**) visual illustration, (**B**) the data to compare the manual and automatic measurement.

In contrast with the previous images indicated in Figure 9, Figure 12 shows 125 manual reading data points. The average difference obtained in this image is 0.14 ± 0.2 mm. This result indicates that the manual measurements may consist of human errors as this is entirely dependent on human visual ability. Furthermore, the ability to reliably calculate these diameters with less systematic errors of a human for a fraction of the time proves this is a useful tool.

This type of algorithm performs poorly on images with high variability in background intensity (i.e., Figure 12) due to the global thresholding techniques used to extract edges. However, using simple image segmentation techniques could enhance the performance of the algorithm and produces accurate results. To avoid this additional step, shadows and large areas of varied intensity in the vicinity of the tube should be minimised. This algorithm is limited by detecting image depth, particularly where the tubes bend towards or away from the camera and the pixels are no longer a consistent unit of measurement. It also underestimates length, not calculating any vertical variation. However, this situation would rarely occur for this type of applications as the samples did not bend towards or away from the camera for substantial distances.

Another main application for this image processing will be in hysterosalpingography (HSG), where a contrast medium is introduced within the reproductive system to evaluate tubal patency. This diagnostic procedure is an essential procedure to understand the potential contribution of this to infertility. This software can assure clinicians if the tubes are patent or not.

## 4. Conclusions

In this paper, a novel vision algorithm measures the biological tubes’ diameter (e.g., fallopian tubes) for further clinical and non-clinical studies. The algorithm has been developed using conventional image processing techniques to detect the fallopian tubes’ irregularity shapes. The data obtained can be useful for diagnosing tubal disorders, such as those related to infertility. Algorithmic extracted diameters are validated by manual measurements using ImageJ software. As the algorithm’s frequency of measurement readings is much higher than the manual measurements, the diameter extraction is more accurate. The programme evaluated four different controlled images and then biological vein images obtained from literature, all of which indicate high algorithm reliability. Different in-house biological tube images were employed to examine the robustness of the algorithm. The algorithm demonstrated high accuracy in the tubes’ visual estimation, high accuracy and repeatability for diameter measurements. This algorithm can be used in various applications to provide accurate diameter estimations for deviating tubes.

Using a conventional image processing technique increases the computational time; however, it is suitable for a low number of accessible images. Artificial intelligence and machine learning techniques are other appropriate routes for diagnostics. However, these require large batches of images for both training level and evaluation.

## Figures and Tables

**Table 1:** The non-maxima gradients along the straight lines and discounted gradients.

Angle	New Angle (°)	Pixels Considered
−22.5 ≤ θ ≤ 22.5 and 157.5 ≤ θ ≤ 202.5	0	P2,P1,P6
22.5 ≤ θ ≤ 67.5 and 202.5≤ θ ≤ 247.5	45	P7,P1,P3
67.5 ≤ θ ≤ 112.5 and 247.5 ≤ θ ≤ 292.5	90	P8,P1,P4
112.5 ≤ θ ≤ 157.5 and 292.5 ≤ θ ≤ 337.5	135	P9,P1,P5

**Table 2:** Comparison between manual and automatic measurements.

Image #	Image Dimensions (PIXEL)	Total along Size Based on Manual Measurements (PIXEL)	Number of Manual Reads	Number of Automatic Reads	Variations (Mean ± SD) Pixel and mm	
A	410 × 304	345.331	16	316	1.59 ± 0.96	0.15 ± 0.09
B	447 × 371	763.689	41	833	5.68 ± 2.16	0.71 ± 0.27
C	520 × 645	1811.579	60	1190	10.11± 6.61	0.81 ± 0.53
D	347 × 372	1000.292	50	958	2.70 ± 1.97	0.37 ± 0.27

## Data Availability

All data and materials supporting the conclusions are described and included in this manuscript.

## Abbreviations

RGB	Red Green Blue
X and Y	The coordinates in 2-dimensions along the Gaussian distribution curve
cdf	cumulative distribution function
Ci	The number pixels at that intensity level
C	total pixel count
Ef	Multiplication factor that optimises the dynamic range
